# Machine Vision Analysis of Ujumqin Sheep’s Walking Posture and Body Size

**DOI:** 10.3390/ani14142080

**Published:** 2024-07-16

**Authors:** Qing Qin, Chongyan Zhang, Mingxi Lan, Dan Zhao, Jingwen Zhang, Danni Wu, Xingyu Zhou, Tian Qin, Xuedan Gong, Zhixin Wang, Ruiqiang Zhao, Zhihong Liu

**Affiliations:** 1 Animal Science Department, Inner Mongolia Agricultural University, Zhaowuda Road, No. 8 Teaching and Research Building, Hohhot 010010, Chinaxx09280506@126.com (D.Z.); olivialovely@foxmail.com (T.Q.); 15547199008@139.com (X.G.);; 2 Key Laboratory of Animal Genetics, Breeding and Reproduction in Inner Mongolia Autonomous Region, Zhaowuda Road, No. 8 Teaching and Research Building, Hohhot 010010, China; 3 Key Laboratory of Mutton Sheep and Goat Genetics and Breeding, Ministry of Agriculture and Rural Affairs, Zhaowuda Road, No. 8 Teaching and Research Building, Hohhot 010010, China; 4 Inner Mongolia Huawen Technology and Information Co., Ltd., Alatan Street, Saihan District Hohhot, Hohhot 010010, China

**Keywords:** sheep, posture, body slanting length, withers height, hip height, chest depth

## Abstract

**Simple Summary:**

Using a neural network approach, this study utilizes Ujumqin sheep as a case study to identify key outline points and calculate posture and body size parameters. The findings reveal that sheep exhibit a more curved knee posture when walking in a channel compared to normal measurement conditions, and tend to lower their heads when passing through passages. This research offers a cost-effective data collection method for studying multi-posture animal husbandry practices.

**Abstract:**

The ability to recognize the body sizes of sheep is significantly influenced by posture, especially without artificial fixation, leading to more noticeable changes. This study presents a recognition model using the Mask R-CNN convolutional neural network to identify the sides and backs of sheep. The proposed approach includes an algorithm for extracting key frames through mask calculation and specific algorithms for head-down, head-up, and jumping postures of Ujumqin sheep. The study reported an accuracy of 94.70% in posture classification. We measured the body size parameters of Ujumqin sheep of different sexes and in different walking states, including observations of head-down and head-up. The errors for the head-down position of rams, in terms of body slanting length, withers height, hip height, and chest depth, were recorded as 0.08 ± 0.06, 0.09 ± 0.07, 0.07 ± 0.05, and 0.12 ± 0.09, respectively. For rams in the head-up position, the corresponding errors were 0.06 ± 0.05, 0.06 ± 0.05, 0.07 ± 0.05, and 0.13 ± 0.07, respectively. The errors for the head-down position of ewes, in terms of body slanting length, withers height, hip height, and chest depth, were recorded as 0.06 ± 0.05, 0.09 ± 0.08, 0.07 ± 0.06, and 0.13 ± 0.10, respectively. For ewes in the head-up position, the corresponding errors were 0.06 ± 0.05, 0.08 ± 0.06, 0.06 ± 0.04, and 0.16 ± 0.12, respectively. The study observed that sheep walking through a passage exhibited a more curved knee posture compared to normal measurements, often with a lowered head. This research presents a cost-effective data collection scheme for studying multiple postures in animal husbandry.

## 1. Introduction

With the advancement of information technology, sheep rearing methods are evolving towards scalability and intelligence. Internet of Things (IoT) technology has been effectively utilized in livestock environmental information, enabling the creation of remote monitoring systems for livestock [1]. The increase in production levels has resulted in a rise in the number of livestock herds. However, data collection and monitoring of livestock present new challenges. Currently, phenotypic assessment of sheep relies mainly on manual measurements. The development of Precision Livestock Farming (PLF) systems, such as automated weighing systems, RFID sensors, and temperature monitoring, has gradually enhanced operational efficiency and farm animal welfare [2].

Research on sheep posture recognition primarily involves accelerometer and visual recognition technologies. Alvarenga et al. classified five exclusive behaviors of sheep herds using accelerometers: grazing, lying down, running, standing, and walking postures [3]. Radeski et al. presented an optimized method for identifying sheep gaits and postures using acceleration values recorded by a triaxial accelerometer [4]. He et al. utilized detection and semantic segmentation to improve sheep weight estimation [5]. However, attaching sensors to sheep’s bodies often induces stress reactions [6]. Non-contact, low-cost, simple, and effective computer vision techniques have been widely applied in animal monitoring processes, significantly contributing to evaluating animal behavior [7].

Mask R-CNN, a versatile model widely used in image segmentation and object detection, offers the advantage of instance segmentation, particularly beneficial in scenarios necessitating the precise delineation of individual objects within an image. In the realm of sheep farming, the utilization of computer vision technology, specifically the Mask R-CNN model, has exhibited potential in monitoring and overseeing sheep behavior and well-being [5,8,9,10,11]. An accurate assessment of sheep body dimensions is vital for evaluating growth status, productivity, and welfare [12]. Prior studies have suggested employing machine vision and deep learning techniques to measure sheep body dimensions, particularly in the standing posture [13,14]. Unlike larger livestock, sheep display a wider range of postures due to their distinct characteristics like additional joints, agility, and complex behaviors [15]. Consequently, there are challenges in effectively utilizing visual methods to capture and analyze various posture states of sheep, including standing, walking, and jumping.

This study utilizes a real-world dataset of segmented sheep from a breeding farm as the foundation for our research. Leveraging the Mask R-CNN convolutional neural network, the study distinguishes between head-down, head-up, and jumping postures of Ujumqin sheep using contour points. It also identifies body size parameters, including head-down and head-up, during the walking state of Ujumqin sheep. This automated approach reduces labor resources for livestock monitoring and provides continuous, real-time data crucial for making informed management decisions. It significantly reduces stress in sheep and the risk of zoonotic diseases, thus ensuring farm animal welfare practices are implemented. This study presents an economical and effective method for collecting data in animal husbandry research.

## 2. Materials and Methods

### 2.1. Experimental Animal

The data for this study were collected from Heshig Animal Husbandry Development Co., Ltd., situated in the East Ujumqin Banner, Inner Mongolia Autonomous Region, China. Established in 1999, the farm is located behind the Urias Mountains, covering an area of 55,000 acres where sheep are raised under grazing conditions. The predominant plant species on the grassland include *Aneurolepididum chinens*, *Artemisia frigida*, *Stipa grandis*, *Stipa krylovii*, and *Xyris pauciflora* Willd. The breeding season for Ujumqin sheep occurs annually in October, with lambing following in February and shearing in June. In August 2023, data collection was carried out to ensure more reliable production data, taking advantage of the mild climate that facilitated sheep production and measurement tasks. To effectively cover different groups, two rounds of herd gatherings were conducted across the region. The herds were randomized and divided into four manageable subgroups. This division was implemented to facilitate image acquisition while preventing large-scale crowding and trampling among the sheep. A total of 1100 Ujumqin sheep aged between 6 months and 5 years were captured with dynamic images, with 43 sheep being excluded due to poor image quality. The images were categorized into two groups: data A and data B. Data A consisted of 548 sheep (241 rams and 307 ewes), and were utilized for posture training, classification parameter adjustment, and constructing a sheep recognition neural network. Each sheep in data A was represented by its best recognition images in head-down, head-up, and jumping poses to facilitate posture classification. The images were further split into training, verification, and test sets in a 7:2:1 ratio, resulting in 7371, 106, and 1053 images, respectively. Data B included 509 Ujumqin sheep, consisting of 211 rams and 298 ewes. The best identification photos of these animals were utilized as a validation dataset to assess the pose analysis model. Furthermore, within data B, the accuracy of manual and machine measurements for body slanting length, withers height, hip height, and chest depth at different postures was evaluated in a subgroup of 285 sheep, comprising 119 rams and 166 ewes.

### 2.2. Collection Process

RFID high-frequency electronic ear tags were affixed to the right ears of lambs at three weeks of age for identification and record-keeping purposes. The sheep were guided through an image data collection channel consisting of a plastic background panel and a rectangular metal frame. Each sheep passed through the channel only once. Two 8-megapixel autofocus high-definition cameras were positioned above and right of the channel. There was plenty of light to observe during the shoot. An industrial computer behind the background panel stored and identified the ear tags. An automated shooting program took images of the sheep’s back and side at 30 frames per second, organizing them into folders named after the ear tag numbers. We manually collected data on gender, ear tag number, and body measurements and compiled it into an Excel spreadsheet for comparative analysis. During manual body measurements, one person restrained the sheep while another collected the data. The sheep were positioned on a flat surface in a natural upright posture, with the head and neck extended and limbs standing upright. Manual measurements were taken for body slanting length, withers height, hip height, and chest depth of the Ujumqin sheep. The measurement standards for body measurements were as follows:

Body slanting length: The distance from the front edge of the shoulder to the rear edge of the ischial tuberosity.

Withers height: The vertical distance from the withers to the ground.

Hip height: The vertical distance from the highest point of the hip joint to the ground.

Chest depth: The straight-line distance from the withers to the lower edge of the sternum.

### 2.3. Experimental Environment

The study utilized KS12A884 cameras equipped with the Sony IMX377 camera sensor model. The cameras were procured from Shenzhen Kingsen Technology Co., Ltd., located in Shenzhen, China. Data storage and transmission considerations were considered, adopting a resolution of 640 × 480. The analysis server was equipped with a central processing unit featuring 36 cores and 72 threads, operating at a base frequency of 2.3 GHz and a boost frequency of 3.7 GHz. Additionally, it included a 32 GB Samsung registered error-correcting code (RECC) memory module sourced from Samsung Electronics, headquartered in Suwon, South Korea and a 2 TB server hard disk for storage sourced from Western Digital, headquartered in San Jose, California, USA. The system operated on the Windows 10 operating system, utilizing the PyTorch framework and Python 3.6 in the software stack. Notably, the learning rate plays a crucial role in the training process, impacting speed and convergence [16]. The network parameters were set to a learning rate of 0.002, a batch size of 8, and 512 epoch iterations to optimize speed and convergence.

### 2.4. Image Preprocessing

**Figure 1:**
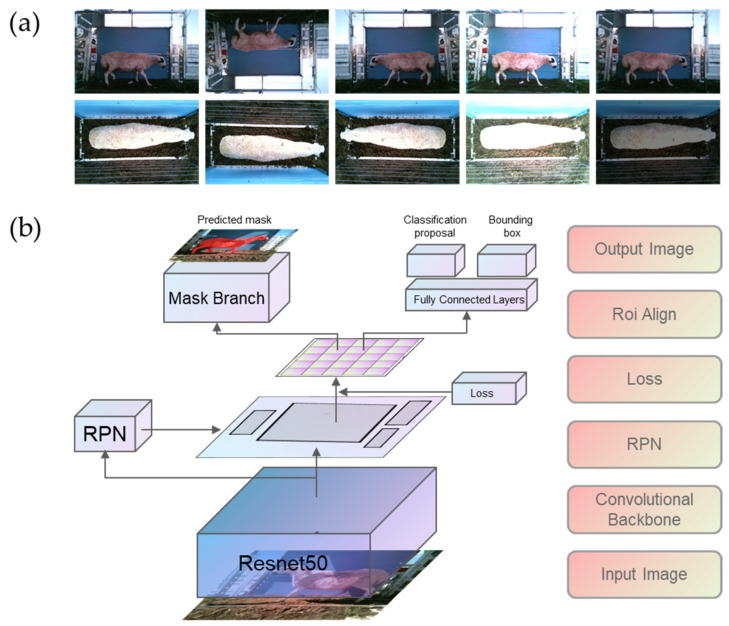
Diagram of image amplification method and recognition model based on Mask R-CNN; (**a**) diagram of image amplification method. image amplification methods include horizontal inversion, vertical inversion, image brightening, and brightness reduction; (**b**) sheep recognition model diagram based on Mask R-CNN; RPN = region proposal network; ROI = region of interest.

This study utilized Mask R-CNN for sheep image recognition, specifically identifying sheep images captured by both back and side cameras. All images underwent manual annotation using the open-source labeling software Labelme version 5.1.1. The quantity of images in the dataset played a crucial role in the detection outcomes. To augment the dataset, a range of techniques were implemented, including horizontal and vertical flipping, as well as adjustments to image brightness through both enhancement and reduction. Figure 1a illustrates the various modes of transformation applied. Ultimately, 10,530 images were collected to train the model, comprising 7470 side images and 3060 back images of Ujumqin sheep. These images constituted the Ujumqin sheep dataset, which was further divided into training, validation, and test sets.

### 2.5. Sheep Recognition Model

The Mask R-CNN model comprises five key components: Input, Backbone network, Region Proposal Network (RPN), Roi Align, and Output. The Input component receives preprocessed image and label data for model training. The Backbone network utilizes ResNet50 as the deep convolutional neural network to extract features from 640 × 480 images, with anchor sizes set to 512 × 512, 256 × 256, 128 × 128, and 64 × 64. This configuration enables the network to recognize low-level details like wool color and texture and high-level features such as sheep position. The feature pyramid network structure combines spatial and semantic information for feature extraction. The RPN filters feature maps by generating varying-sized boxes based on aspect ratios. Roi Align resamples feature maps to a uniform size for classification and regression tasks. Ultimately, the Output component yields class, sheep position, and sheep mask. Figure 1b presents a diagrammatic representation of the sheep recognition model architecture, grounded in the Mask R-CNN framework.

### 2.6. Key Frame Screening

**Figure 2:**
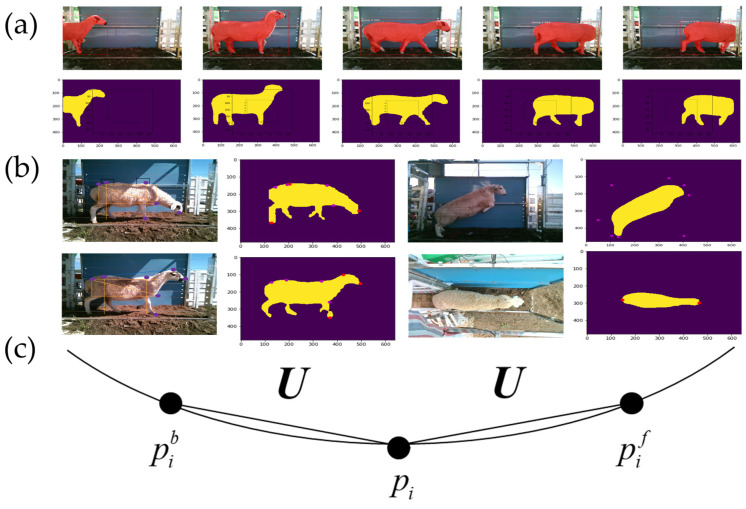
Sheep feature point recognition results and key frame recognition results under different postures; (**a**) key frame identification map based on the mask layer; (**b**) resulting diagram of sheep feature point recognition under different postures; (**c**) neighborhood diagram supported by U chord length curvature.

To identify key frames of sheep passing through the channel, this study utilized the Mask R-CNN approach for sheep object detection. This method facilitated the recognition of sheep positions in the images by overlaying a mask on top of them. Data on sheep areas could be extracted by applying binary thresholding to the processed images. During the passage of sheep, a multitude of images were captured. However, for effective recognition, it was imperative to select images displaying clear and complete contours of the sheep, ideally with the entire body centered in the frame. This study employs the method of region division and the number of image mask pixels to identify key frames. Figure 2a presents a diagram of the key frame screening used for the movement of Ujumqin sheep.

(1) $$Area=n_{i}\sum_{i=1}^{3}R_{i}$$

where *R*_1_ is located at (1/3x–2/3x, 0.375–0.625y), *R*_2_ is located at (1/6x–5/6x, 1/4y–3/4y), and *R*_3_ is located at (x, y), x represents the X-axis resolution and y represents the Y-axis resolution. The weighting coefficients *n_i_* were assigned as follows: *n*_1_ = 10, *n*_2_ = 0.5, and *n*_3_ = 0.01.

### 2.7. Sheep Posture Recognition

During the study, it was observed that sheep exhibit a specific posture only when jumping, characterized by the bending of their legs and the movement of their head upwards. This results in a distinct angle formed by the minimum bounding rectangle, which is not seen during normal movement. The diagonal angle between the minimum bounding rectangle and the minimum rectangle effectively captured the sheep’s body curvature and leg extension, enhancing the accuracy of posture information during jumping. The study utilized Python to calculate the contour points of both rectangles, followed by statistical analysis using SPSS version 22 to compare posture variations. A decision tree method was also employed to identify key parameters for classifying sheep jumping behavior. A neural network was then utilized to detect the sheep mask, enabling the assessment of the sheep’s highest point and head position for posture analysis. Sheep images were segmented into four regions (top-left, bottom-left, top-right, and bottom-right) based on the highest point and head position. For instance, if the highest point is in the top-right and the head is above the hip, the sheep is categorized as looking upward; whereas if the highest point is in the top-right but the head is below the hip, the sheep is classified as being in a lowered position.

### 2.8. Sheep Body Sizes Recognition

Due to the potential instability of sheep skeletons during jumping, this study did not factor body measurements into jumping states. For sheep postures characterized by lowered and raised heads, the ConvexHull function of OpenCV2 was utilized to pinpoint contour points. The calculation methods used for body slanting length and chest depth in various walking postures were consistent, whereas those used for withers height and hip height varied.

The computation methodology for body slanting length and chest depth was as follows. The study employed a recursive algorithm-based approach to extract the maximum inscribed rectangle within a spatial domain for calculating these parameters. Specifically, the maximum circumscribed rectangle was initially identified to define the spatial region, and then recursive algorithms were utilized to identify multiple inscribed rectangles within this region. Subsequently, the areas of these inscribed rectangles were calculated and represented in a list format. Leveraging the properties of a stack, the maximum rectangular area was identified. The diagonal positions of the maximum inscribed rectangle denoted the feature points for body slanting length, and the distance of chest depth was the distance between the feature point of withers height and the feature point to the right of the body slanting length. The recognition of different postures is shown in Figure 2b.

Hip in raised head posture: The ConvexHull function was utilized to calculate the convex hull points of the sheep contour, identifying the rightmost overlapping point within the sheep contour as the feature point for withers height.

Withers height in raised head posture: Line A is defined as the line connecting the top of the sheep’s head to the shoulder point. All contour points on line A were selected, and the distance between each contour point and line A was computed. The point with the longest distance was selected as the feature point for withers height.

Withers height and hip height in lowered head posture: In the lowered head posture, the sheep’s scapula and hip bones protrude, identifying feature point locations for withers height and withers height. The distance between the sheep’s body’s left and right boundary points is denoted as S. The withers height range is defined as 3/8S–4/8S, and the withers height range is 6/8S–7/8S. The U-shaped chord length curvature algorithm demonstrates strong noise resistance and rotational invariance, meeting the specified criteria. Therefore, this algorithm is employed to calculate the curvature. By utilizing the U-shaped chord length curvature, the point with the highest curvature was determined as the feature point for withers height and hip height in the lowered head posture. Figure 2c is a neighborhood diagram that was supported by the U-shaped chord length curvature.

(2) $$c_{i}=s_{i}\sqrt{1-{\left(\frac{D_{i}}{2U}\right)}^{2}}$$

where $s_{i}=sign\left[\left(x_{i}-x_{i}^{b}\right)\left(y_{i}^{f}-y_{i}^{b}\right)-\left(x_{i}^{f}-x_{i}^{b}\right)\left(y_{i}-y_{i}^{b}\right)\right]$, $D_{i}=||p_{i}^{b}p_{i}^{f}||$. $\left(x_{i},y_{i}\right)$, $\left(x_{i}^{b},y_{i}^{b}\right)$, and $\left(x_{i}^{f},y_{i}^{f}\right)$ are the coordinates of $p_{i}$, $p_{i}^{b}$, and $p_{i}^{f}$, respectively and U is the setting constant.

### 2.9. Loss Function

The loss function of Mask R-CNN comprises three main components: classification loss, bounding box regression loss, and mask segmentation loss. These elements are combined to create the comprehensive loss function utilized during model training.

(3) $$L=L_{\mathrm{cls}}+L_{\mathrm{box}}+L_{\mathrm{mask}}$$

$L_{cls}$ is the classification loss, which penalizes the model for errors in category prediction. $L_{box}$ is the bounding box regression loss, which measures the difference between predicted bounding boxes and ground truth boxes. $L_{mask}$ is the mask segmentation loss function, which quantifies the accuracy of minimizing the discrepancy between predicted masks and ground truth masks. This study assessed the outcomes of the Mask R-CNN model on the validation set. Once the loss function ceased to decrease significantly, it was determined that the model had been adequately trained and was identified as the best model. Subsequently, the precision and recall metrics were calculated.

(4) $$\mathrm{Precision}=\frac{TP}{TP+FP}$$

(5) $$\mathrm{Recall}=\frac{TP}{TP+FN}$$

where *TP* is the true positive number, *FP* is the false positive number, and *FN* is the false negative number. Precision denotes the precision of identifying positive samples, while recall indicates the proportion of correctly identified positive samples out of all positive samples.

### 2.10. Actual Distance Conversion

Accurately measuring sheep body size required converting pixel distances to actual distances. Due to the disparity between the camera coordinate system and the real-world coordinate system, pixel distances from the camera had to be converted into centimeter measurements for precise sheep body dimensions. The Euclidean formula was utilized for this conversion. To ensure accuracy, the back height of Ujumqin sheep was limited to 90 cm, with the back calibration plate moving within a range of 0 to 90 cm and the side calibration plate within a range of 0 to 50 cm, with an image acquisition interval of 5 cm. This study adopted a multivariate regression approach for pixel-to-centimeter conversion calculations at different camera distances to address varying proportional relationships at different distances. By using the known actual length of the calibration plate, the ratio of pixel values to actual centimeters was considered the dependent variable, while the distance from the camera to the sheep and the distance from the sheep to the distance channel were treated as independent variables for polynomial regression.

## 3. Results

### 3.1. Neural Network Training Results

**Figure 3:**
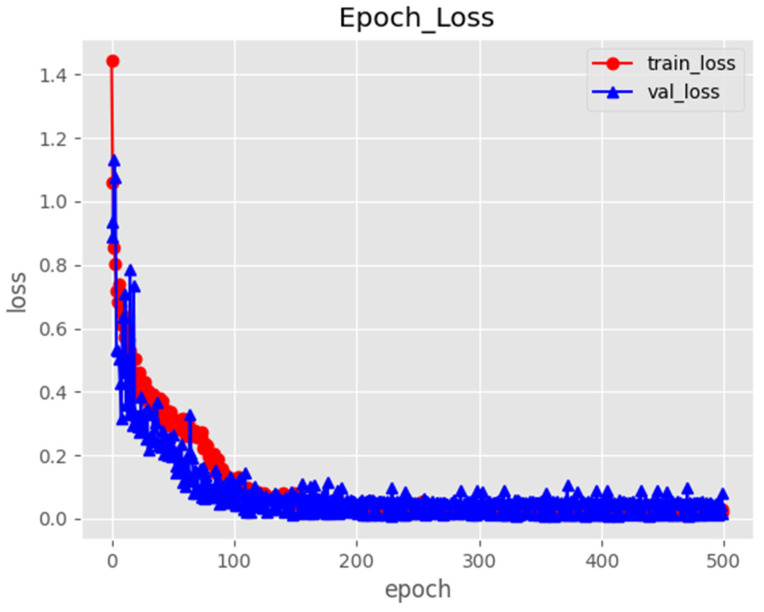
Loss curve of Mask R-CNN model.

The training data analysis revealed a rapid decrease in the model’s loss value within the initial 100 training rounds, indicating a swift improvement in the model’s performance during the early learning stage. Subsequently, from the 100th to the 200th round, the rate of the reduction in the loss value slowed down, suggesting the model was approaching a local optimal solution. Beyond 200 training rounds, the loss value stabilized, signaling that the model’s performance had converged, rendering further training unlikely to yield significant enhancements. This marks the optimal point to cease training. The 200th epoch was identified as the most optimal model in this research. The loss curve of the Mask R-CNN model is shown in Figure 3. To assess the recognition accuracy of Ujumqin sheep using the Mask R-CNN model, 1053 images were tested. The precision of the study is 0.99, with a recall result of 0.01, indicating the model’s ability to accurately classify back and side images of Ujumqin sheep.

### 3.2. Sheep Posture Recognition Results

The study examines the relationship between the posture of sheep and the angles of the minimum area rectangle and its diagonal. Table 1 showed that the angle varies between different postures, with the jumping position exhibiting the highest angle deviation. We documented the number and angle of sheep in various postures, with 309 sheep in the lower head position with an angle of 2.84 ± 3.64, 76 sheep in the head-up position showing an angle of 3.16 ± 2.45, and 163 sheep in the jumping position displaying an angle of 15.44 ± 8.35. There was no significant difference in the angle between the minimum area rectangle and the diagonal of the minimum rectangle in the head-down and head-up states. However, when sheep jumped, their bodies exhibit a rising posture, resulting in a significantly higher angle than the head-down and head-up states. The study utilized decision tree results, with a value exceeding 8.9 serving as a parameter for identifying the jumping posture in subsequent validation experiments.

A total of 509 Ujumqin sheep images were analyzed, with 482 images being correctly identified, resulting in an overall accuracy rate of 94.70%. The ewes exhibited more head-down and jumping behaviors than rams while crossing the aisle. The proportions of head-down, head-up, and jumping in rams were 65.36%, 18.05%, and 16.59%, respectively, while ewes also showed the same proportions of 66.12%, 15.46%, and 18.42%, respectively. Variations in the recognition accuracy were noted across different behavioral categories, with a higher accuracy observed in head-down and head-up behaviors and a slightly lower accuracy in jumping behavior, as shown in Table 2. For rams, 125 out of 134 instances of lower head behavior were correctly identified (93.28% accuracy), while all 37 instances of head-up behavior were correctly identified (100% accuracy). In contrast, 30 out of 34 jumping behaviors were correctly identified, resulting in an accuracy rate of 88.24%. Among ewes, 196 out of 201 instances of head-down and head-up state behavior were correctly identified (97.51% accuracy), 45 out of 47 instances of head-up behavior were correctly identified (95.74% accuracy), and 49 out of 56 jumping behaviors were correctly identified, with an accuracy of 87.50%.

### 3.3. Body Size Accuracy Results

**Figure 4:**
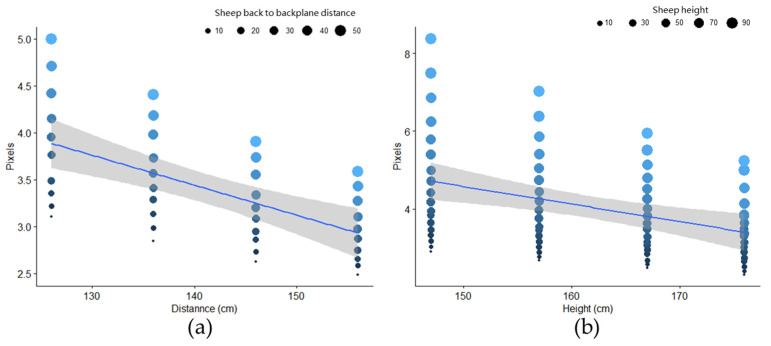
The pixel fitting map of the side camera and the back camera at different distances. (**a**) The pixel fitting map of the side camera at different distances; (**b**) the pixel fitting map of the back camera at different distances. Abbreviations: Trends are denoted by blue lines, with the gray areas indicating the 95% confidence interval of the trend line.

Pixel fit maps of the lower back camera and the side camera at various distances are depicted in Figure 4. The figure illustrates a linear change in the scale conversion relationship of the camera. At the same distance between the camera and the channel backplane in Figure 4a, the closer the sheep was to the backplane, the greater the camera conversion relationship. In Figure 4b, when the position of the back camera in relation to the ground was the same, the higher the height of the sheep, the greater the conversion relationship of the camera.

(6) $$y_{\mathrm{side}}=92.67+(0.05795*x_{1})-(0.0008332*(x_{1}^{2}))+(0.000006949*(x_{1}^{3}))-(1.529*x_{2})+(0.008718*(x_{2}^{2}))-(0.00001699*(x_{2}^{3}))$$

(7) $$y_{\mathrm{back}}=-40.38+(0.01818*x_{3})+(0.0001908*(x_{3}^{2}))+(0.000001377*(x_{3}^{3}))+(1.035*x_{4})-(0.007941*(x_{4}^{2}))+(0.00001959*(x_{4}^{3}))$$

where $y_{\mathrm{side}}$ and $y_{\mathrm{back}}$ are the coefficient between the pixel distance of the side camera and the actual distance of the back camera, respectively. *x*_1_ indicates the distance from the back of the sheep to the channel backplane, and *x*_2_ indicates the distance from the camera to the backplane. *x*_3_ indicates the height of the sheep photographed from the side, and *x*_4_ indicates the distance from the camera to the ground.

Statistics were collected for the body slanting length, withers height, hip height, and chest depth measurements of 285 Ujumqin sheep, as outlined in Table 3. When the sheep had their heads positioned low, the machine measurements showed higher values for body slanting length and chest depth compared to manual measurements, while withers height and hip height exhibit the opposite trend. Conversely, in the head-up position, except for hip height, the machine measurements yielded higher values than the manual measurements. The errors for the head-down position of rams, in terms of body slanting length, withers height, hip height, and chest depth, were recorded as 0.08 ± 0.06, 0.09 ± 0.07, 0.07 ± 0.05, and 0.12 ± 0.09, respectively. For rams in the head-up position, the corresponding errors were 0.06 ± 0.05, 0.06 ± 0.05, 0.07 ± 0.05, and 0.13 ± 0.07, respectively. The errors for the head-down position of ewes, in terms of body slanting length, withers height, hip height, and chest depth, were recorded as 0.06 ± 0.05, 0.09 ± 0.08, 0.07 ± 0.06, and 0.13 ± 0.10, respectively. For ewes in the head-up position, the corresponding errors were 0.06 ± 0.05, 0.08 ± 0.06, 0.06 ± 0.04, and 0.16 ± 0.12, respectively. Chest depth exhibits the most significant error, while hip height displays the smallest error.

## 4. Discussion

The present study focused on analyzing the movement patterns of Ujumqin sheep. The Mask R-CNN convolutional neural network model was utilized to detect the contour points of sheep, allowing for the analysis of body size traits under various postures. These traits included body slanting length, withers height, hip height, and chest depth. Previous research by Bene et al. has shown that sheep exhibit different postures during exercise, emphasizing the impact of exercise conditions on their postures [17]. Current studies on sheep behavior primarily involve accelerometers and visual recognition technology. For example, Alvarenga et al. used accelerometers to categorize five distinct behaviors of sheep, such as herding, lying down, running, standing, and walking [3]. Radeski et al. proposed an optimized method for identifying sheep gait and posture by analyzing acceleration values from a triaxial accelerometer [4]. Gu et al. introduced a deep learning-based approach for detecting sheep behaviors, including standing, eating, and lying down [18]. Nonetheless, these methods encounter challenges related to sheep wearables and computational detection performance limitations.

Zhang et al. highlight the significance of analyzing the minimum area of a rectangle by investigating the directional characteristics of a patch obtained from the aspect ratio of the rectangle [19]. Chaudhuri et al. utilized minimum boundary rectangles to extract various features, such as the aspect ratios of the longer and shorter axes, offering a method to scrutinize sheep’s jumping posture using minimum area and minimum rectangles [20]. Similarly, Sant’ana et al. employed the minimum rectangular area approach to assess the physical indicators of sheep [21]. This study involved analyzing sheep’s posture as they moved through a channel by inputting contour point data, utilizing the angle between two rectangles and the key point positions during the sheep’s walking state, achieving an overall accuracy rate of 94.70%. The recognition accuracy for head-down and head-up states exceeded 90%, while that for jumping posture surpassed 85%. Xu et al. utilized Mask R-CNN to detect two typical behaviors, standing and lying down, of varying group sizes, achieving an accuracy of 94% in the validation set [8], similar to the results obtained in this study. Polk et al.‘s research showed that sheep exercising on an inclined treadmill exhibited a more bent knee posture compared to those on a horizontal treadmill, indicating that sheep bend their knees to move swiftly during fast walking [22]. This study also noted underestimations in withers height and hip height in the head-down state.

Utilizing advanced technologies such as machine learning and visual image analysis is crucial for improving body size measurement and posture analysis in animals [23]. Witte et al. highlighted the significance of visual assessment in discerning the quality disparities between segmentation masks [24]. Zhao et al. employed Mask R-CNN to achieve a 93.7% accuracy in detecting Hu sheep with an IoU threshold of 0.5 [25]. Xu et al. investigated the behavior of sheep standing and lying down in pens of varying sizes, achieving over 94% accuracy in the validation set [8]. The model’s classification accuracy in this study was high, possibly attributable to the custom dataset used. The classification of side and back images of sheep was distinct, with a large sample size contributing to the elevated accuracy.

Zhang et al. proposed a non-contact method using machine vision to measure the body size of small-tailed Han sheep, aiming to overcome the limitations of manual measurement [13]. Study on Alpagota goats demonstrated that a dual-camera recognition system accurately predicted withers height, chest depth, and body length with a 3.5% error rate [26]. Similarly, in Ujumqin sheep, visual image measurements exhibited a 5% error in predicting body slanting length, withers height, and hip height compared to manual measurements, with a 10% error in chest depth [14]. Various factors contribute to errors in body size calculations, including differences between machine-generated image data and human measurements. Animals must be in a standardized posture for accurate measurement. Manual measurements also introduce variability due to factors like the experience level of personnel, procedural deviations, and fatigue from prolonged work. The presence of long chest hair during movement can lead to neural networks misinterpreting hair as part of the body, resulting in significant errors in chest depth calculations. Lina et al. emphasized the challenges of accurately measuring animal body size parameters, highlighting issues such as postural changes and the impact of features like chest hair [27]. This was also mentioned by Mathis et al. [28], illustrating that there are still challenges with current vision technology. While neural networks excel in processing visible images, they face limitations when dealing with coat and tail fat interference. Future studies should investigate the use of multiple cameras to address these challenges. Moreover, developing a user-friendly interface and integrating it with existing farm management tools is crucial to promoting farmers’ adoption of such systems.

As the agricultural landscape continues to evolve globally, the integration of automation and intelligent technologies becomes increasingly crucial. This study utilized a neural network model to calculate the contour points of Ujumqin sheep, enabling the estimation of pose and body size parameters. The automated system developed in this research reduces the possibility of human oversight and significantly enhances the efficiency of farm management. Moreover, the real-time body size monitoring feature offers data that empower farmers to make swift decisions regarding feeding and health management, ultimately enhancing the overall well-being of the animals. This study accurately identified sheep’s posture and body size and laid the groundwork for the development of strategies for predicting health status. This research aligns with the growing emphasis on precision and intelligence in modern agriculture, presenting a cost-effective method for collecting data to investigate various animal husbandry practices across different postures.

## 5. Conclusions

The study developed a Mask R-CNN recognition model to identify the side and back views of Ujumqin sheep accurately. The model achieved a posture classification accuracy of 94.70%. This paper investigated the impact of different sheep postures, such as head-down, head-up, and jumping, on the measurement accuracy of key body size parameters. The study observed that sheep walking through a passage exhibited a more curved knee posture than normal measurements, often with a lowered head. These findings emphasize the importance of considering the impact of sheep posture on body size measurement accuracy. The proposed data collection scheme, utilizing the Mask R-CNN-based recognition model, offers a cost-effective solution for studying multiple postures in animal husbandry. This research contributes to developing more accurate and reliable methods for assessing sheep body size.

## Figures and Tables

**Table 1:** Statistics of sheep in different postures.

Postures	N	Angles
head-down	309	2.84 ± 3.64 ^a^
head-up	76	3.16 ± 2.45 ^a^
jump	163	15.44 ± 8.35 ^b^

Different lowercase letters indicate statistically significant differences between the postures (*p* < 0.05). Abbreviations: The angle is formed by the minimum area rectangle and the minimum rectangle diagonal.

**Table 2:** Table of statistics for the recognition of Ujumqin sheep under different behaviors.

Gender	Postures	N	Correctly Identified	Accuracy
ram	head-down	134	125	93.28%
	head-up	37	37	100.00%
	jump	34	30	88.24%
ewe	head-down	201	196	97.51%
	head-up	47	45	95.74%
	jump	56	49	87.50%
Total		509	482	94.70%

**Table 3:** Comparison of machine measurement accuracy between Ujumqin rams and ewes head-down and head-up posture.

Postures	N	Gender	Types	BSL	WH	HH	CD
head-down	91	ram	M	71.63 ± 9.79 (13.67%)	61.91 ± 8.38 (13.54%)	65.51 ± 7.66 (11.7%)	30.96 ± 4.19 (13.54%)
			A	69.91 ± 6.39 (9.14%)	65.61 ± 6.92 (10.54%)	68.57 ± 6.54 (9.53%)	29.57 ± 4.56 (15.43%)
			Acc	0.08 ± 0.06	0.09 ± 0.07	0.07 ± 0.05	0.12 ± 0.09
	127	ewe	M	68.81 ± 8.52 (12.38%)	59.02 ± 8.33 (14.11%)	63.18 ± 7.49 (11.85%)	29.51 ± 4.16 (14.11%)
			A	67.33 ± 5.98 (8.89%)	62.1 ± 5.23 (8.42%)	65.31 ± 5.81 (8.89%)	27.92 ± 3.76 (13.48%)
			Acc	0.06 ± 0.05	0.09 ± 0.08	0.07 ± 0.06	0.13 ± 0.1
head-up	28	ram	M	67.47 ± 8.82 (13.07%)	64.29 ± 7.18 (11.17%)	62.37 ± 6.97 (11.17%)	31.08 ± 3.76 (12.08%)
			A	67.2 ± 6.15 (9.15%)	63.3 ± 5.69 (9%)	65.53 ± 5.86 (8.94%)	27.71 ± 3.61 (13.04%)
			Acc	0.06 ± 0.05	0.06 ± 0.05	0.07 ± 0.05	0.13 ± 0.07
	39	ewe	M	67.4 ± 6.9 (10.23%)	64.45 ± 7.45 (11.57%)	65.26 ± 7.32 (11.22%)	32.22 ± 3.73 (11.57%)
			A	66.78 ± 5.6 (8.38%)	62.45 ± 4.67 (7.48%)	65.4 ± 4.67 (7.14%)	28.09 ± 3.68 (13.11%)
			Acc	0.06 ± 0.04	0.08 ± 0.06	0.06 ± 0.04	0.16 ± 0.12

Abbreviations: BSL = body slanting length; WH = withers height; HH = hip height; CD = chest depth; N = number; M = machine measurement; A = manual measurement; Acc = accuracy.

## Data Availability

The original contributions presented in the study are included in the article. Further inquiries can be directed to the corresponding author(s).
